# Structure of a hexameric form of RadA recombinase from *Methanococcus voltae*

**DOI:** 10.1107/S1744309112010226

**Published:** 2012-04-20

**Authors:** Liqin Du, Yu Luo

**Affiliations:** a Department of Biochemistry, University of Saskatchewan, 107 Wiggins Road, Suite A3, Saskatoon, Sasktchewan S7N 5E5, Canada

**Keywords:** RadA recombinase, *Methanococcus voltae*

## Abstract

Archaeal RadA proteins are close homologues of eukaryal Rad51 and DMC1 proteins and are remote homologues of bacterial RecA proteins. For the repair of double-stranded breaks in DNA, these recombinases promote a pivotal strand-exchange reaction between homologous single-stranded and double-stranded DNA substrates. This DNA-repair function also plays a key role in the resistance of cancer cells to chemotherapy and radiotherapy and in the resistance of bacterial cells to antibiotics. A hexameric form of a truncated *Methanococcus voltae* RadA protein devoid of its small N-terminal domain has been crystallized. The RadA hexamers further assemble into two-ringed assemblies. Similar assemblies can be observed in the crystals of *Pyrococcus furiosus* RadA and *Homo sapiens* DMC1. In all of these two-ringed assemblies the DNA-interacting L1 region of each protomer points inward towards the centre, creating a highly positively charged locus. The electrostatic characteristics of the central channels can be utilized in the design of novel recombinase inhibitors.

## Abstract 2

Hexameric rings of RadA recombinase from *M. voltae* have been crystallized. Structural comparisons suggest that homologues of RadA tend to form double-ringed assemblies.

## 1. Introduction

Bacterial RecA (Clark & Margulies, 1965 ▶), archaeal RadA (Sandler *et al.*, 1996 ▶) and eukaryal Rad51 (Shinohara *et al.*, 1992 ▶) and DMC1 (Bishop *et al.*, 1992 ▶) proteins form a superfamily of recombinases (also called DNA strand-exchange proteins; Seitz & Kowalczykowski, 2000 ▶). Homologous recombination appears to be essential in the repair of double-stranded DNA breaks and the restarting of stalled replication forks (Cox, 1998 ▶; Cox *et al.*, 2000 ▶; Courcelle *et al.*, 2001 ▶; Lusetti & Cox, 2002 ▶; Kowalczykowski, 2000 ▶). These proteins facilitate a pivotal DNA strand-exchange process between a single-stranded DNA (ssDNA) and a homologous double-stranded DNA (dsDNA) in homologous recombination. Electron-microscopic and crystallo­graphic results have revealed strikingly similar ‘active’ recombinase assemblies in the form of right-handed helical filaments with approximately six monomers per turn (VanLoock *et al.*, 2003 ▶; Conway *et al.*, 2004 ▶; Wu *et al.*, 2004 ▶; Chen *et al.*, 2008 ▶; Sheridan *et al.*, 2008 ▶; Li *et al.*, 2009*a* ▶). The milestone structures of *Escherichia coli* RecA (EcRecA) in complex with a series of DNA molecules have shed light on the exact mechanism of homologous DNA strand exchange (Chen *et al.*, 2008 ▶). Crystallized ‘inactive’ filaments with shorter helical pitches have also been observed (Story *et al.*, 1992 ▶). Ring-shaped forms with 6–8 protomers have also been commonly observed by electron microscopy (Heuser & Griffith, 1989 ▶; Yu & Egelman, 1997 ▶; Passy *et al.*, 1999 ▶; Yang *et al.*, 2001 ▶; Galkin *et al.*, 2006 ▶; McIlwraith *et al.*, 2001 ▶; Masson *et al.*, 1999 ▶). Only heptameric rings of *Pyrococcus furiosus* RadA (PfRadA) and octameric *Homo sapiens* DMC1 (HsDMC1) have previously been crystallized (Shin *et al.*, 2003 ▶; Kinebuchi *et al.*, 2004 ▶). A reconstructed hexameric EcRecA model has been derived from electron microscopy (Yu & Egelman, 1997 ▶). In addition to the three commonly found forms, crystal structures of overwound three-monomer-per-turn filaments (Ariza *et al.*, 2005 ▶) and left-handed filaments of *Sulfolobus solfataricus* RadA (SsRadA; Chen *et al.*, 2007 ▶; Chang *et al.*, 2009 ▶) have also been observed. Here, we report the first crystal structure of hexameric RadA from *Methanococcus voltae* devoid of its first 60 amino-acid residues (Δ_60_MvRadA). Crystal-packing analysis and comparison with the heptameric PfRadA structure and the octameric HsDMC1 structure indicated that these proteins can form two-ringed assemblies.

## 2. Experimental procedures

### 2.1. Cloning, protein preparation and crystallization

The open reading frame of residues 61–322 of RadA from *M. voltae* was inserted between the *Nde*I and *Xho*I sites of pET28a (Novagen). The resulting plasmid was verified by DNA sequencing using T7 promoter and terminator primers. The recombinant Δ_60_MvRadA was overexpressed in *E. coli* BL21 Rosetta2 (DE3) cells (Novagen) at 310 K for 4 h using 0.5 m*M* isopropyl β-d-1-thiogalactopyranoside as the inducer. The cells were disrupted by sonication. The insoluble particles were removed by centrifugation at 12 000*g*. Soluble proteins were first separated by nickel-affinity chromatography. The polyhistidine tag was then removed by overnight digestion with 1:100(*w*:*w*) thrombin (Sigma–Aldrich) at 294 K. Gel-filtration chromatography was performed with a Sephacryl S-300 HR column (GE Healthcare) using a buffer composed of 0.5 *M* sodium acetate and 30 m*M* Tris–HCl pH 7.9. The purified protein was concentrated to ∼30 mg ml^−1^ by ultrafiltration.

### 2.2. Crystallization of Δ_60_MvRadA and diffraction data collection

Δ_60_MvRadA crystals (space group *C*2) were grown by the hanging-drop method and grew to maximum dimensions of 0.4 × 0.3 × 0.2 mm. The optimal well solution consisted of 33% polyethylene glycol 400, 1.0 *M* NaNO_3_, 50 m*M* MES–NaOH buffer pH 6.7 and 0.06% thymol. A crystal was transferred into the well solution, looped out of the solution and frozen in a nitrogen cryostream at 100 K. The diffraction data set was collected and processed using a Bruker PROTEUM R system at the Saskatchewan Structural Sciences Centre (at a wavelength of 1.5418 Å). The statistics of the diffraction data are listed in Table 1 ▶.

### 2.3. Structural determination and refinement

The previously solved RadA model (PDB entry 1t4g; Wu *et al.*, 2004 ▶) was used as the search model for molecular replacement using *Phaser* (McCoy *et al.*, 2007 ▶). Six monomers were located in the asymmetric unit, which is consistent with the existence of noncrystallographic sixfold rotational symmetry. The model was iteratively rebuilt using *XtalView* (McRee, 1999 ▶) and refined using *CNS* (Brünger *et al.*, 1998 ▶) and *REFMAC* (Murshudov *et al.*, 2011 ▶). Statistics of the refinement and model geometry are given in Table 1 ▶. 90.6% of nonglycine residues fell in the most favoured region of the Ramachandran plot. No residues were found in the disallowed region. The electron-density map was generated by *Coot* (Emsley & Cowtan, 2004 ▶) and rendered by *Raster*3*D* (Bacon & Anderson, 1988 ▶). The ribbon and electrostatic surface figures were rendered using *CCP*4*MG* (Potterton *et al.*, 2004 ▶). C^α^ traces were generated by *MolScript* (Kraulis, 1991 ▶) and *Raster*3*D*. The coordinates and structure factors have been deposited in the Protein Data Bank (Bernstein *et al.*, 1977 ▶; Berman *et al.*, 2000 ▶, 2003 ▶) with code 4dc9.

## 3. Results

### 3.1. The overall structure of a hexameric form of Δ_60_MvRadA

**Figure 1:**
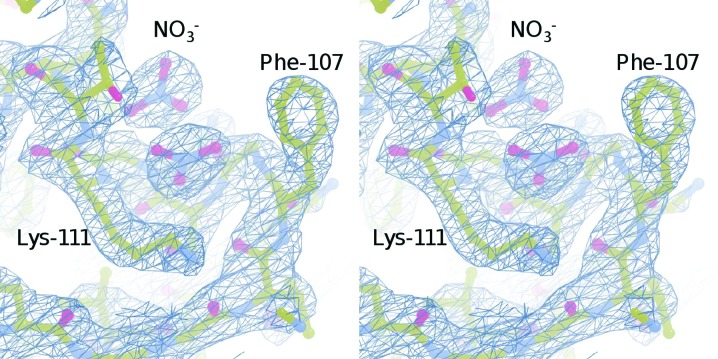
Electron-density map of the P-loop. The final σ-weighted 2*F*_o_ − *F*_c_ map contoured at 1.2σ is shown in stereo. Two putative nitrate-binding sites are also shown. C, N and O atoms are shown in yellow, blue and red, respectively.

**Figure 2:**
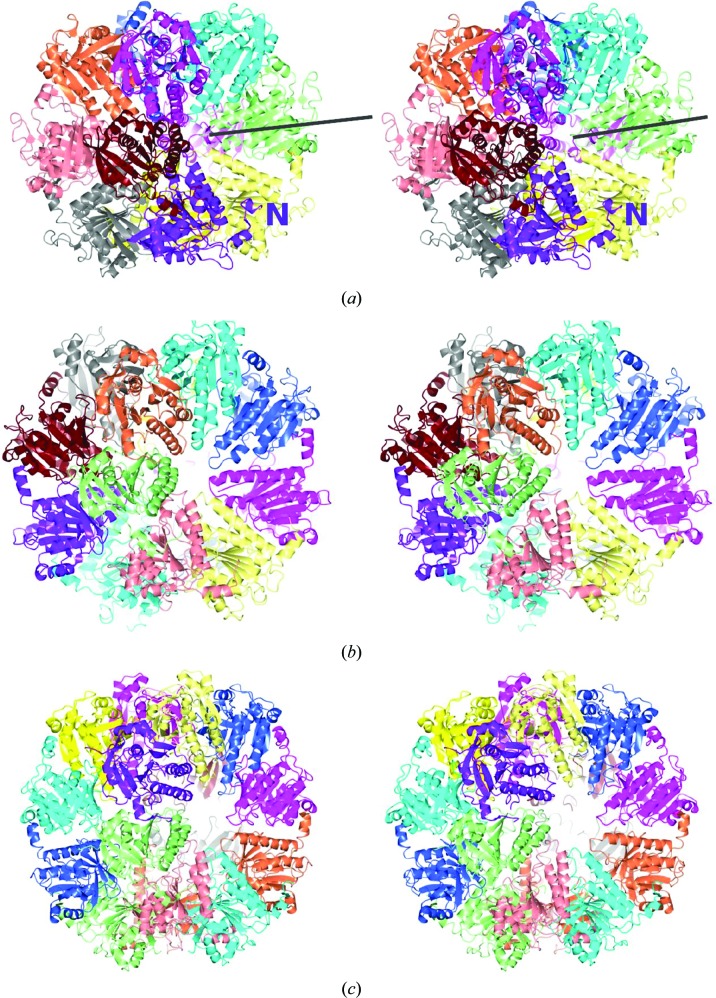
Two-ringed assemblies of RadA and DMC1. Ribbon representations are shown in stereo. The ribbons are coloured by chain. (*a*) Δ_60_MvRadA hexamers. Residue 60 of one subunit is labelled ‘N’. The central sixfold axis is marked by a dark line. (*b*) PfRadA heptamers. The N-terminal domain of the PfRadA structure is omitted. (*c*) HsDMC1 octamers.

As in RecA orthologues, a polymerization motif centred at Phe74 contributes to the oligomerization of MvRadA (Wu *et al.*, 2004 ▶). In order to test the effect of this motif on the oligomerization of MvRadA, we made a series of truncation mutants of MvRadA that lacked 60–65 N-terminal residues. Similar to the previously studied Δ_62_MvRadA (Galkin *et al.*, 2006 ▶), Δ_60_MvRadA is active in hydrolyzing ATP in the presence of poly(dT)_36_ but inactive in promoting DNA strand exchange (data not shown). The crystal structure of Δ_60_MvRadA was solved by the molecular-replacement method. The ATP-binding P-­loop (residues Gly105–Thr112) was ordered with two putative nitrate ions (Fig. 1 ▶), consistent with the requirement for a high concentration of sodium nitrate in the crystallization solution. The peptide chain was largely ordered except for residues 261–268 in the DNA-interacting L2 region (residues Asn256–Arg285). The six monomers of Δ_60_MvRadA formed a closed ring with approximate sixfold rotational symmetry (Fig. 2 ▶ *a*). The central channel is lined by L1 regions (residues Arg218–Arg230) and has a diameter of 10 Å.

### 3.2. Two-ringed assembly

Crystal-packing analysis as well as the self-rotation function indicated that the Δ_60_MvRadA hexamers further packed into face-to-face two-ring assemblies with *D*6 point-group symmetry (Fig. 2 ▶ *a*), with one of the six twofold axes coinciding with the crystallographic twofold axis. During the gel-filtration stage of the purification of the RadA proteins, the full-length MvRadA as well as the truncated protein eluted predominantly as a species with a molecular weight of around 200 kDa. As such, the Δ_60_MvRadA protein is likely to exist as single rings in solution.

### 3.3. Conformational change of MvRadA

**Figure 3:**
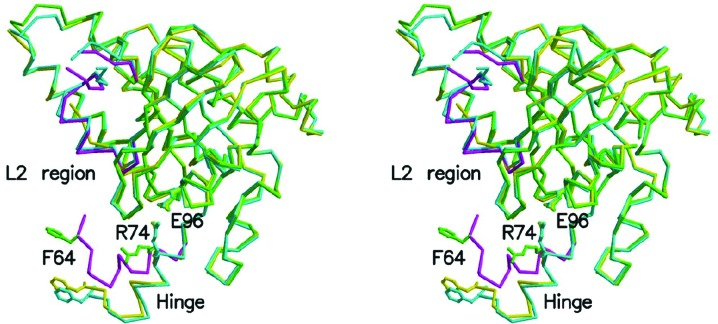
The conformational changes of MvRadA. Three C^α^ traces are shown in stereo. The Δ_60_MvRadA structure is shown in green, except for its C^α^ trace from 61 to 75 and from 256 to 285 (magenta). The previously determined ATPase-active filament of MvRadA is shown in cyan. The Δ_62_MvRadA structure in the inactive filament is shown in yellow.

The Δ_60_MvRadA protein is composed solely of the conserved ATPase domain found in RecA orthologues. This domain starts with a polymerization motif centred around a hydrophobic residue (Phe64 in MvRadA) which protrudes into a hydrophobic pocket in an adjacent monomer in the recombinase polymer. In comparison with the previously determined ATPase-active filament (104–105 Å pitch) of MvRadA (PDB entry 2fpm; Wu *et al.*, 2005 ▶; Qian *et al.*, 2005 ▶) and the Δ_62_MvRadA filament (PDB entry 2gdj) with a shorter pitch (91 Å; Galkin *et al.*, 2006 ▶), residues 61–75 showed the most noticeable translation (Fig. 3 ▶). This region also contains a conserved Arg74 residue which has been shown to be important for the conformational flexibility of RadA and Rad51 (Chen *et al.*, 2007 ▶). In all previously determined filamentous structures of RadA from *M. voltae* (Wu *et al.*, 2004 ▶, 2005 ▶; Qian *et al.*, 2005 ▶, 2006 ▶, 2007 ▶; Galkin *et al.*, 2006 ▶; Li *et al.*, 2009*b* ▶) and *M. maripaludis* (MmRadA; Li *et al.*, 2009*a* ▶), this Arg74 residue forms a salt bridge with Glu96 (yellow and cyan structures in Fig. 3 ▶). In the hexameric Δ_60_MvRadA structure the side chain of Arg74 was observed in a noticeably different conformation (green side chain in Fig. 3 ▶) that is incapable of retaining the salt bridge. Two recurrent conformations have been observed in the previously determined MvRadA and MmRadA helical structures. One (cyan structure in Fig. 3 ▶) is largely ordered except for residues 261–268 in the L2 region and is likely to correspond to the ATPase-active conformation (Wu *et al.*, 2005 ▶). The other (yellow structure in Fig. 3 ▶) is more disordered in the L2 region and is likely to correspond to the ‘inactive’ post-ATP hydrolysis conformation (Qian *et al.*, 2005 ▶). The conformation of each RadA monomer in the hexameric form (green and magenta structures in Fig. 3 ▶) clearly resembles the ATPase-active form (cyan structure in Fig. 3 ▶). A short helix (residues Gly275–Ala282) was observed in the L2 region which corresponds to helix G in EcRecA (Story *et al.*, 1992 ▶; De Zutter *et al.*, 2001 ▶).

### 3.4. Similar assemblies of PfRadA and HsDMC1

**Figure 4:**
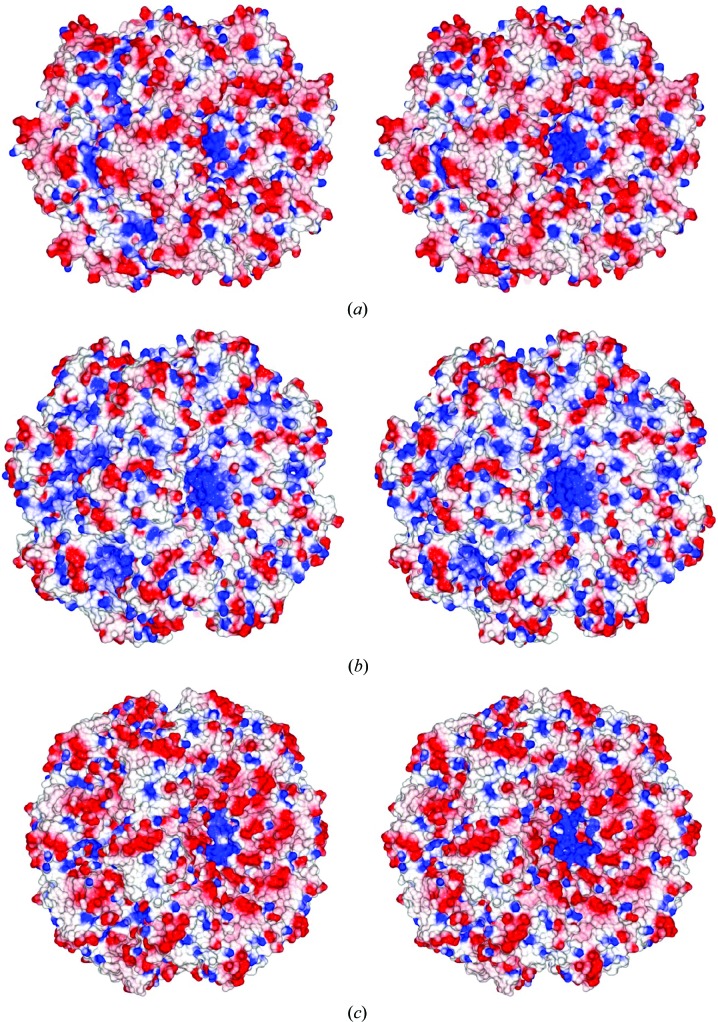
Electrostatic properties of RadA and DMC1. The solvent-accessible surfaces of two-ringed assemblies are shown in stereo. The negatively charged area is coloured red, while the positively charged area is coloured blue. (*a*) Δ_60_MvRadA hexamers. (*b*) PfRadA heptamers. (*c*) HsDMC1 octamers.

Interestingly, the crystal packing of heptameric PfRadA (Shin *et al.*, 2003 ▶; PDB entry 1pzn) and octameric HsDMC1 (Kinebuchi *et al.*, 2004 ▶; PDB entry 1v5w) suggests that they both form similar two-ringed assemblies with *D*7 and *D*8 point-group symmetry (Figs. 2*b* ▶ and 2*c* ▶), respectively. These assemblies resemble the face-to-face double rings observed for HsDMC1 and SsRadA in the presence of dsDNA by electron microscopy (Passy *et al.*, 1999 ▶; Masson *et al.*, 1999 ▶; Yang *et al.*, 2001 ▶). In all such assemblies the L1 regions (residues Arg218–Arg230 of MvRadA) line a central channel. Each L1 region has three conserved arginine residues (Arg218, Arg224 and Arg230 in MvRadA) in RadA/Rad51/DMC1 proteins. As a result, the central channels of such assemblies are highly positively charged (the central blue regions in Fig. 4 ▶).

## 4. Discussion

Unlike the structures of filamentous MvRadA, the hexameric Δ_60_MvRadA structure revealed a different conformation of Arg74 which is no longer capable of retaining the salt bridge to Glu96. As such, the crystal structure of Δ_60_MvRadA further supports the notion that the residue equivalent to Arg74 of MvRadA modulates the conformational changes which give rise to flexibility in the protein assemblies of orthologous proteins (Chen *et al.*, 2007 ▶).

The conformational similarity of Δ_60_MvRadA and the ATPase-active form of MvRadA in the helix G region suggests that this short helix is inherently stable. In the structures of filamentous MvRadA and MmRadA disorder of helix G has been correlated with either the presence of ADP or the absence of proper cationic bridging between the C-terminal carbonyl groups of helix G and the γ-phosphate of the ATP analogue. This structural feature of Δ_60_MvRadA is consistent with the notion that ATP hydrolysis in recombinase filaments triggers disorder of helix G and the larger L2 region (Qian *et al.*, 2005 ▶; Li *et al.*, 2009*a* ▶).

We recently observed that polyanionic compounds such as metatungstate could inhibit MvRadA (Li *et al.*, 2009*b* ▶) by competing with DNA for positively charged L1 regions lined along an axial groove in the MvRadA filament. Although there is no evidence that such two-ringed assemblies exist in solution in the absence of DNA, their highly cationic cavities suggest that anionic compounds that replace DNA could stabilize such recombinase assemblies and thus inhibit the formation of active recombinase filament. It has been discovered that tumour cells tend to have an elevated level of Rad51 expression, which correlates with their resistance to radiotherapy and chemotherapy (Klein, 2008 ▶). Therefore, a Rad51 inhibitor could serve as a potential adjuvant for cancer therapy. In addition to suppressing the ATPase activity (Wigle *et al.*, 2006 ▶, 2009 ▶; Wigle & Singleton, 2007 ▶; Sexton *et al.*, 2010 ▶), mimicking the polymerization motif (Cline *et al.*, 2007 ▶) and blocking the DNA-binding groove in the recombinase filament (Li *et al.*, 2009*b* ▶), the two-ringed assemblies of RadA and DMC1 proteins suggest a fourth strategy for inhibiting the recombinase activities of RecA orthologues.

## Supplementary Material

PDB reference: RadA recombinase, 4dc9

## Figures and Tables

**Table 1:** Data-collection and refinement statistics Values in parentheses are for the highest resolution shell.

Data collection	
Space group	*C*2
Unit-cell parameters (Å, °)	*a* = 186.35, *b* = 118.58, *c* = 141.73, α = γ = 90, β = 138.05
Resolution (Å)	39.2–2.60 (2.69–2.60)
*R*_merge_	0.070 (0.293)
〈*I*/σ(*I*)〉	8.5 (2.6)
Completeness (%)	90.5 (90.1)
Unique reflections	57374 (5768)
Multiplicity	3.6 (3.1)
Refinement	
Resolution (Å)	30–2.6
No. of reflections	54446
*R*_work_/*R*_free_	0.206/0.266
No. of atoms	12092
Protein	11982
Ligand/ion	48
Water	62
*B* factors (Å^2^)	55.7
Protein	55.7
Ligand/ion	43.8
Water	38.5
R.m.s. deviations	
Bond lengths (Å)	0.013
Bond angles (°)	1.70
